# T-cell response to checkpoint blockade immunotherapies: from fundamental mechanisms to treatment signatures

**DOI:** 10.1042/EBC20220247

**Published:** 2023-09-28

**Authors:** Thomas A.E. Elliot, David A.J. Lecky, David Bending

**Affiliations:** Institute of Immunology and Immunotherapy, College of Medical and Dental Sciences, University of Birmingham, Birmingham, U.K.

**Keywords:** Anti-tumour immune responses, Immunotherapy, T cell exhaustion, T cell receptor signalling, T cells

## Abstract

Immune checkpoint immunotherapies act to block inhibitory receptors on the surface of T cells and other cells of the immune system. This can increase activation of immune cells and promote tumour clearance. Whilst this is very effective in some types of cancer, significant proportions of patients do not respond to single-agent immunotherapy. To improve patient outcomes, we must first mechanistically understand what drives therapy resistance. Many studies have utilised genetic, transcriptional, and histological signatures to find correlates of effective responses to treatment. It is key that we understand pretreatment predictors of response, but also to understand how the immune system becomes treatment resistant during therapy. Here, we review our understanding of the T-cell signatures that are critical for response, how these immune signatures change during treatment, and how this information can be used to rationally design therapeutic strategies. We highlight how chronic antigen recognition drives heterogeneous T-cell exhaustion and the role of T-cell receptor (TCR) signal strength in exhausted T-cell differentiation and molecular response to therapy. We explore how dynamic changes in negative feedback pathways can promote resistance to single-agent therapy. We speculate that this resistance may be circumvented in the future through identifying the most effective combinations of immunotherapies to promote sustained and durable antitumour responses.

## Introduction

Activation of T cells is controlled by their T-cell receptor (TCR), which recognises short amino acid sequences (peptides) presented on major histocompatibility complex (MHC) molecules by antigen-presenting cells (APCs). Conventional T cells can be CD8^+^ ‘cytotoxic’ or CD4^+^ ‘helper’ subsets, defined by their ability to bind peptide-loaded MHC (pMHC) class I or II, respectively. Direct recognition of pMHC on target cells is important for killing [1]; MHC I is constitutively expressed and loaded with self-peptide in almost all cells, whereas MHC II expression is constitutive only in ‘professional’ APCs such as dendritic cells (DCs). As such, many cancers are MHC II^−^ [2], making CD8^+^ T cells the primary cytotoxic population driving the anticancer response. However, in some cases, cells of epithelial lineage can express MHC II in response to interferon signalling [3] and cytotoxic CD4^+^ T cells can kill MHC II^+^ cancer cells [4].

T-cell activation results in production of key cytokines, such as Interferon-gamma (IFNγ) and tumour necrosis factor-alpha (TNF-α), and cytolytic granules such as Perforin and Granzymes. Activated T cells proliferate and can differentiate into memory cells, persisting for long periods of time primarily in the blood and lymphoid tissues, primed to respond quickly to subsequent infection or malignancy. Alternatively, activated T cells can differentiate into an effector phenotype – effectors are more cytotoxic and can reside in tissues to exert their effector function, but retain less proliferative capacity.

Strength of TCR signalling has important roles in T-cell differentiation and function. T cells need to distinguish between weak tonic signals that occur in the lymphoid environment to promote survival [5,6], and strong, foreign antigen signals that require an immune response. In mice, comparatively stronger TCR signalling promotes effector function over memory in both CD4^+^ [7] and CD8^+^ T cells [8–10] during acute stimulation. Also, whilst cytotoxic fate of CD8^+^ T cells is largely independent of initial signal strength, strength of TCR recognition on target cells dictates directional polarisation of cytolytic granules and effective killing [11,12].

## Effective antitumour immunity requires T-cell responses to tumour neoantigens

Tumours are derived from self, making their recognition challenging for T cells that are self-tolerised during thymic development [13]; nevertheless, T cells can recognise a variety of cancer associated patterns through their TCR [14], the most potent of which are neoantigens. Neoantigens are novel amino acid sequences arising from nonsynonymous mutations that can deliver a signal to the TCR when presented on MHC molecules [15]. High numbers of nonsynonymous mutations tend to be associated with exogenous mutagens or certain types of genetic instability [16]. Accordingly, experimental induction of high neoantigen burden via loss of DNA mismatch repair machinery results in tumour rejection by mice [17].

In addition to key effector functions, TCR ligation drives the expression of multiple costimulatory and coinhibitory receptors that can recruit kinases or phosphatases to positively or negatively regulate TCR signalling [18]. Programmed cell death 1 (PD-1) appears to be the dominant coinhibitory receptor and is expressed by both naïve and antigen experienced T cells upon TCR ligation. Inhibitory signalling via PD-1 targets both the CD28 costimulatory molecule [19,20] and the TCR itself [21]. Other coinhibitory receptors, such as lymphocyte-activation gene 3 (LAG-3), T-cell immunoreceptor with Ig and ITIM domains (TIGIT), and T-cell immunoglobulin and mucin domain 3 (TIM-3), are expressed only in T cells with more prolonged antigen experience due to epigenetic inaccessibility of their loci in naïve populations [22]. Signalling via coinhibitory receptors raises the TCR signalling threshold, such that an antigenic stimulus that would otherwise activate a T cell fails to elicit a response [23]. Blockade of coinhibitory receptors, or their ligands, using therapeutic monoclonal antibodies (immune checkpoint blockade (ICB)) can increase T-cell function and drive clinical benefit in some types of cancer [24].

In the U.K., NICE approval for ICB therapy is based on evidence of improved survival from clinical trials. Response to ICB is highly variable between different types of cancer. Cancers with high mutation rates, such as melanoma and lung squamous-cell carcinoma, tend to respond well [25]. However, even within patient groups that respond well, such as melanoma, there are some patients that are therapy resistant. Therapy resistance can broadly be defined as primary resistance, where patients fail to respond to primary treatment, and acquired resistance, where patients initially respond but then become resistant [26].

Much of what drives resistance is tumour intrinsic [27]. Analysis of genetic signatures in pretreatment tumour biopsies has revealed predictors of response to ICB, such as clonal mutational burden and signatures of immune recruitment such as CXCL9 expression [27]. In patients that do initially respond, ICB exerts immune pressure on the tumour, to which it can often adapt. Tumours can undergo immunoediting by acquisition of mutations in key antigens that are driving the immune response, or the pathways that present these antigens to T cells. However, in some cases, patients may still respond to ICB in the absence of antigen recognition. For example, in β2-Microglobulin-mutated dMMR colorectal cancer where γδ T cells recognise stress ligands on tumour cells via NKG2D [28].

Tumours may also acquire mutations in JAK/STAT signalling pathways that mediate the response to IFNγ signalling [29], an important effector mechanism of the anticancer CD8 T-cell response. Tumours can remodel their microenvironment to promote therapy resistance [30]; factors such as hypoxia [31], immune exclusion by tumour stroma [32], and infiltration of regulatory immune cells such as myeloid-derived suppressor cells [33] can subvert the anticancer immune response.

Distinct from tumour intrinsic factors, there are T-cell intrinsic factors that can also mediate resistance. Understanding the T-cell signatures (or biomarkers) that predict response to therapy, but also how these signatures change during therapy can provide insight into mechanisms of therapy resistance. Experimental work in animal models of chronic antigen exposure can help us understand the fundamental processes that govern T-cell regulation, and therefore T-cell intrinsic therapy resistance. Here, we discuss the fundamental mechanisms that regulate T-cell biology in the face of chronic antigen exposure. We overview how preclinical mouse models have set a platform for translational discovery and highlight key similarities and differences that have been observed in human clinical studies of patients on ICB.

## Negative feedback control of T cells in mouse models of infection and tolerance

Experimental models of chronic antigen exposure, such as lymphocytic choriomeningitis virus (LCMV) infection, and techniques to identify antigen-specific T cells such as TCR transgenics and pMHC tetramers, have informed our fundamental understanding of negative feedback control of T cells.

Negative feedback mechanisms, including coinhibitory receptors, are an intrinsic and indispensable feature of T-cell function; their absence can cause activation-induced T-cell death [34] and lethal immunopathology during infection [35]. Chronic antigen exposure in cancer or infection can drive ‘exhaustion’ – a state of T-cell differentiation distinct from effector/memory phenotypes, characterised by expression of multiple coinhibitory receptors and progressive loss of function [36,37]. Exhausted T cells lose proliferative potential, long-term survival, ability to produce cytokines, and ability to kill target cells. Although there are many similarities between exhausted CD8^+^ T cells and effectors arising from acute antigen stimulus, there are transcriptional signatures and characteristics specific to T-cell exhaustion, uncoupled from effector function [38]. In mice, blockade of the ligand for PD-1 (PD-L1), can induce proliferation in LCMV specific exhausted CD8^+^ T cells [35].

LCMV studies have highlighted the significant heterogeneity in T-cell exhaustion (Table 1). Many have drawn a distinction between T cells that are exhausted (T_EX_, usually indicated by coexpression of multiple coinhibitory receptors including PD-1 and Tim-3 or CD39 [36]), and the precursor to these cells (T_PEX_, usually indicated by coexpression of PD-1 and transcription factor T-cell factor 1 (TCF1) [39] or CXCR5 [40,41]). Loss of T-cell functionality in response to chronic antigen is graded and T_PEX_ cells are at an early point in the trajectory. T_PEX_ cells can undergo a TCR signal-dependent conversion to T_EX_ cells [42], a process that is augmented by T-cell intrinsic IFNγ signalling [43]. In contrast, T_EX_ cells are a stable population that maintain phenotypic and functional features in transfer experiments [44]. In many ways, T_PEX_ and T_EX_ demonstrate a functional polarisation like memory and effector CD8^+^ T cells during acute stimulation. T_PEX_, unlike T_EX_, show a selective ability to undergo proliferative burst following ICB [39,40].

**Table 1:** Phenotypic and functional differences between naive and exhausted subsets of CD8^+^ T cells during chronic LCMV infection in mice

	Naive	T_PEX_	T_EX_ intermediate	T_EX_ terminal
TCF1	++	+	-	-
TOX	-	+	++	++
TBET	-	+	-	-
EOMES	-	-	+	+
CD127	+	+/-	-	-
CD62L	++	+/-	-	-
CX3CR1	-	-	+	-
CXCR6	-	-	-	+
PD-1	-	+	++	++
TIM-3	-	-	+	+
KLRG1	-	-	+	-
Proliferative capacity	+	+	-	-
Cytotoxicity	-	-	+	+/-
Cytokine production	+	+	+	-

Transcription factors such as TOX [34,45,46], BLIMP-1 [47], MYB [48], NR4A1 [49,50], and NFAT [51] mediate the exhaustion programme by controlling the expression of negative regulators of TCR signalling. Exhausted T cells also undergo significant epigenetic rewiring following as little as 5 days of chronic antigen exposure [52,53]. In mouse models, it is largely thought that terminally exhausted T cells are incapable of response to ICB due to the epigenetic repression of genes involved in important T-cell functions [54] and *TCF7* (encoding TCF1) itself [55].

Failure of T_EX_ cells to respond to ICB monotherapy may be a result of functional redundancy between numerous coinhibitory receptors [56]. In contrast, negative signalling via PD-1 may be the limiting factor for T_PEX_ cells to signal via their TCR, explaining their selective response to monotherapy. This may then suggest that blockade of multiple coinhibitory receptors is necessary to restore signalling in T_EX_. For example, it has been shown that dual blockade of coinhibitory receptors PD-1 and TIGIT can restore cytokine production in TOX^+^ CD8^+^ tumour-infiltrating lymphocytes (TILs) *in vitro* [57].

Some studies have challenged this idea. For example, combining blockade of PD-L1 with a vaccination approach has been shown to expand PD-1hi antigen-specific CD8^+^ T cells in an infection model [58]. Additionally, TIM-3^+^ exhausted T cells can proliferate in response to engineered Interleukin-10 [59]. These data show it may be possible to reinvigorate terminally exhausted T cells. Discrepancies between studies may be due to functional heterogeneity within TCF1^−^-exhausted subsets. Some TCF1^−^ subsets are at an intermediate stage of exhaustion, they express CX3CR1 and KLRG1 and make IFNγ. Other more terminally exhausted cells express CXCR6 and produce very low levels of IFNγ [60].

Not just duration, but TCR signal strength appears to be a key determinant of fate decisions among exhausted CD8 T-cell subsets. T cells with higher avidity for antigen, as determined by tetramer staining, showed a biased for terminal exhaustion differentiation [60]. Additionally, utilising the *Nr4a3*-Tocky reporter of TCR signalling and a model of adaptive tolerance, we have highlighted CD4^+^ T-cell transcriptional programmes that are related to defined TCR signal strengths [23]. Crucially, we found that strong TCR signalling increased the expression of many coinhibitory receptors, in agreement with work on CD8^+^ T cells in tumour models [61]. Additionally, we have found significant overlap in genes associated with strong TCR signalling, and genes up-regulated in antigen experienced T cells reactivating in response to PD-1 blockade. This evidence suggests that blockade of inhibitory signalling does not only increase the probability that a given antigen-experienced T cell will reactivate in response to subsequent TCR signals, but that the qualitative strength of the signal is higher. This agrees with *in vitro* studies that have shown selective sensitivity to PD-1-mediated inhibition in genes that require strong TCR signalling [62].

## Negative feedback control of T cells in mouse models of cancer

Chronic infection models have some key limitations in understanding T-cell negative feedback in cancer. Tumour-reactive T cells circulate between the tumour, blood, and lymphoid tissue and a systemic immune response is key for effective anticancer immunity [63]. In contrast with systemic LCMV infections, tumour antigens are largely restricted to the tumour site. This is an important consideration, as cellular therapy studies have found withdrawal from chronic antigen stimulation can restore function to exhausted T cells [64].

Many paradigms from LCMV studies have been replicated in mouse models of cancer. Subcutaneous implantation of tumour cells leads to generation of T_EX_ and T_PEX_ cells that are transcriptionally analogous to those generated during LCMV infection [42]. T_PEX_ are essential for the maintenance of a T-cell response in mouse models of cancer, they can traffic between lymph nodes and tumours [65,66], acting as a reservoir [67] to supply tumours with cells that gain effector functions upon arrival [68]. Despite representing a small proportion of tumour-specific CD8^+^ T cells, selective deletion of tumour-specific *Tcf7*^+^ CD8^+^ T cells accelerates tumour growth and diminishes response to ICB in mouse models [69]. These data in mouse models suggest that signatures relating to T_PEX_ may indicate an effective ongoing anticancer immune response, and the presence of cells that can respond well to ICB therapy.

**Figure 1:**
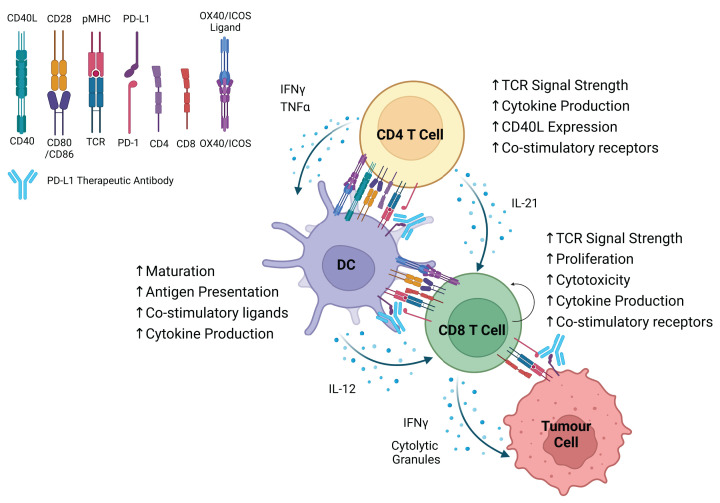
Graphical representation of reported changes to function of CD4^+^ T cells, CD8^+^ T cells, and DCs in response to αPD-L1 immunotherapy Distinct from direct effect on CD8^+^ T cells, immunotherapy can act via CD4^+^ T cells and DCs to support CD8^+^ T-cell function via cytokine production and costimulation.

T-cell exhaustion is not limited to CD8^+^ T cells. Cancer neoantigens are frequently restricted to MHC II and recognised by CD4^+^ T cells in both mice [70] and humans [71]. In a poorly immunogenic mouse model of cancer, experimental induction of an MHC I restricted neoantigen only conferred sensitivity to ICB when an MHC II restricted neoantigen was also induced [72]. CD4^+^ T-cell recognition of tumour antigen led to increased numbers of CD8^+^ T cells through the well-established principle of CD4^+^ T-cell help [73], whereby CD4^+^ T cells provide support to CD8^+^ T cells in the form of costimulation and cytokine production, either directly or via DCs (Figure 1). This happened despite lack of MHC II expression on tumour cells, indicating that CD4^+^ T cells recognised tumour antigens that were phagocytosed and presented by professional APCs. Therefore, whilst direct recognition of MHC II^+^ tumour cells can mediate tumour rejection [74], it is not prerequisite for CD4^+^ T-cell involvement in the anticancer immune response.

Like work in models of chronic infection and adaptive tolerance, strength of TCR signalling can dictate fate of antitumour CD8^+^ T cells. High-affinity interactions between the TCR and pMHC drive higher levels of negative feedback, whilst particularly low affinity interactions lead to ineffective killing [61]. TCR signal strength appears to be a key determinant of T_EX_ versus T_PEX_ fate; whilst both subsets can share clonotypes [68], it appears that lower-affinity TCR-pMHC interactions favour T_PEX_ differentiation [75].

Expression levels of coinhibitory receptors are dependent on TCR signal strength and PD-1 blockade increases TCR signal strength [23,60]. We have observed elevated expression of genes encoding multiple coinhibitory receptors in antigen-adapted T cells that reactivated their TCR signalling in response to PD-1 blockade [23]. In mouse models of cancer, it has been observed that T cells bound by therapeutic PD-1 antibodies have increased expression of TIM-3 [76]. Consequently, combination of PD-1 and TIM-3 blockade showed better clinical response than PD-1 blockade alone [76,77]. Additionally, PD-L1 and LAG-3 combination therapy has shown synergistic effects on CD4^+^ and CD8^+^ T-cell cytokine production and tumour control [78]. Therefore, combination blockade is an attractive strategy to not only target T cells with very high levels of inhibitory signalling but also to combat therapy induced dynamic feedback and acquired therapy resistance [79]. Signatures that can identify dynamic changes in negative feedback may therefore be valuable in the design of combinatorial ICB therapies and on-therapy monitoring of efficacy.

## T-cell signatures in response to ICB in human cancer

Mouse models are a valuable tool for studying the processes that underlie ICB therapeutic success. However, the time periods for disease development differ significantly, and many mouse models are unable to account for the significant interpatient heterogeneity in human cancer.

### Systemic response to ICB

Whilst many studies in mouse models of cancer focus on intratumoural T cells, it is now recognised that an effective response to ICB is in fact systemic [63]. Interaction between T cell PD-1 and DC PD-L1 in tumour-draining lymph nodes is an important facet of ICB activity in mice [80]. In melanoma patients, ICB can lead to clonal replacement of intratumoural CD8^+^ T cells with those from peripheral sites [81]. In addition, sequencing of peripheral blood T cells before ICB therapy has found that expanded cytotoxic CD8 T-cell clones are associated with beneficial response [82]. The peripheral blood of melanoma patients exhibits an increased number of highly expanded clones as compared with healthy controls, reflective of T-cell proliferation in response to recognition of tumour antigens [82]. An increase in the number of highly expanded clones in response to therapy is associated with good clinical outcomes [83]. Clones possessing a central memory signature can convert to a cytotoxic effector signature in the peripheral blood of patients responding to ICB [82]. This demonstrates how ICB can increase TCR signal strength to drive differentiation of cytotoxic effector cells to mediate tumour cell killing. These findings are corroborated by analysis of tumour reactive T cells from peripheral blood of lung cancer patients [84]: successful ICB responses were associated with an increase in expression of effector/exhaustion markers and a decrease in memory phenotypes during treatment.

In the periphery, highly expanded cytotoxic CD8 T-cell clones show the greatest transcriptional response to ICB, without increasing their clonal size [82]. These cells express many genes typically associated with CD8 T-cell exhaustion such as *TOX*, *HAVCR2*, and *TIGIT*. Similar studies in urothelial cancer have shown expanded tumour-specific CD8 clones with a cytotoxic phenotype in the periphery are predictive of effective ICB response [85]. This marks a clear difference between mouse LCMV models and human disease-exhausted CD8^+^ T cells in human cancer are likely not ‘nonresponders’ but respond differently to CD8^+^ T cells at an earlier stage of differentiation. This highlights a mechanism distinct from proliferation as a key determinant of response to ICB and agrees with mouse *in-vitro* studies that have found exhausted CD8^+^ T cells to be effective killers [61].

### Intratumoural responses to ICB

Early studies in humans receiving ICB identified TIL intrinsic signatures of response, including increased CD8 T-cell infiltration, T-cell proliferation, IFNγ signalling, and granzyme B production [86]. Tumour antigen-specific CD4^+^ T cells can accumulate within human cancers [87], exhibiting hallmarks of exhaustion such as coexpression of multiple inhibitory receptors (including CD39), loss of cytokine production (a key mechanism for helping CD8^+^ T cells), and transcriptional similarities to TOX^+^ CD8^+^ T cells [88]. Exhausted CD4^+^ T cells can respond to immunotherapy: blockade of PD-1 during *in-vitro* stimulation of patient-derived PD-1^+^ CD39^+^ CD4^+^ TILs led to increased cytokine production and expression of the costimulatory ligand CD40L. Following pretreatment with anti-PD-1, coculture of exhausted CD4^+^ TILs with autologous DCs and CD8^+^ T cells led to DC priming and CD8^+^ T-cell proliferation [88]. These studies highlight the fact that both CD4^+^ and CD8^+^ T cells can lose critical antitumour functions, following chronic antigen stimulation and are both targets of ICB (Figure 1). Indeed, we recently cross-referenced transcriptional signatures of ICB in melanoma patients with those found in mouse CD4 T cells receiving high-strength TCR signals. We identified a five gene signature associated with clinical response, highlighting increased TCR signal strength as a key factor in driving patient survival. We termed this gene signature TCR.strong (*ICOS, TNFRS4* (OX40), *STAT4, TNIP3, IRF8*) [23]. Elevated expression of ICOS and OX40 suggests that the ICB-mediated increase in TCR signal strength acts to increase costimulation via inducible receptors (Figure 1).

A study of human lung cancer TILs utilised sequencing of complimentary determining region 3 (CDR3) loci to determine TCR specificity and cross-referenced to scRNA-seq data [84]. This allowed transcriptional comparisons of TILs reactive for tumour neoantigens and the numerous bystanders [89] reactive for resolved (influenza) or latent (EBV) infections. Tumour antigen-reactive TILs contained features of mouse T_EX_ and were enriched for gene signatures associated with tissue residency (*CD103, HOBIT*), effector function (*GZMB, IFNG*), negative feedback (*HAVCR2, ENDTP1* (CD39)), and transcriptional control of exhaustion (*TOX, BLIMP1*). Accordingly, they also expressed low levels of genes associated with memory. Similar results have been shown in melanoma [90].

Transcriptional comparisons of tumour-reactive TILs between patients found that signatures associated with memory (*IL7R, TCF7*) were correlated with a good clinical response, whereas many genes associated with effector function, exhaustion, and tissue residency were associated with treatment resistance [84]. This highlights that whilst most TILs have an exhausted signature, it is atypical TILs – resembling descriptions of T_PEX_ – that are crucial for effective therapy responses. In support of this notion, T_PEX_ can be sustained within intratumoural niches [91] to promote survival, and the presence of intratumoural TCF1^+^ PD-1^+^ CD8 T cells is predictive of a positive response to ICB in melanoma patients [90,92].

### T-cell signatures of exhaustion and therapy resistance

**Figure 2:**
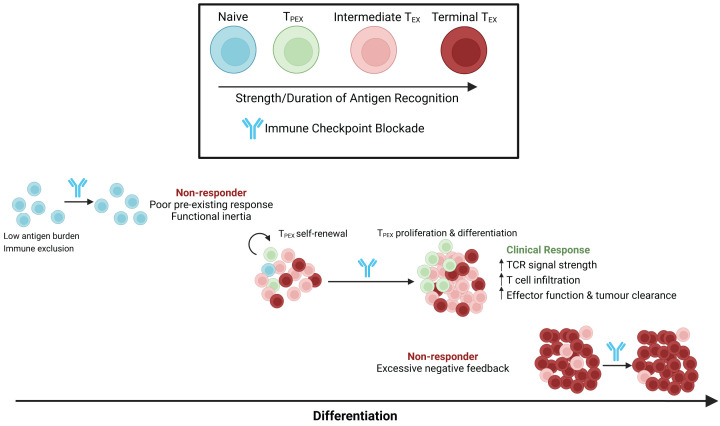
Graphical representation showing how αPD-L1 immunotherapy can modulate intratumoural CD8^+^ T-cell differentiation during treatment Ineffective response may be a result of poor pre-existing response or previous terminal differentiation. Presence of less-differentiated T_PEX_ cells allows for proliferative burst and differentiation, driving clinical benefit.

T-cell signatures from human studies suggest that in addition to a weak pre-existing response, homogenous exhaustion to a strong pre-existing response can also drive primary therapy resistance (Figure 2). These data suggest it may be possible to avoid primary resistance by early intervention with ICB, prior to homogenous T-cell exhaustion. Treatment with ICB in the neoadjuvant setting has led to improved survival over adjuvant treatment in melanoma [93] and can drive pathological responses in canonical ICB nonresponders such as pMMR colorectal cancer [94].

ICB-induced dynamic negative feedback may contribute to acquired therapy resistance in humans. In the peripheral blood of melanoma patients responding to ICB, CD8 T cells that proliferate in response to ICB increase their expression levels of PD-1 and CTLA-4 [95]. This is not a peripheral phenomenon, since other studies have highlighted signatures of increased intratumoural negative feedback following ICB including expression of PD-1 and LAG-3 [96], and V-domain immunoglobulin suppressor of T-cell activation (VISTA) [97]. Increases in TCR strong signature following ICB were higher in immunotherapy naive patients as compared with those who progressed following CTLA-4 blockade [23]. This suggests that ICB can lead to enhanced negative feedback, driving acquired resistance to future ICB therapy. In support of this, new combination approaches that target coinhibitory receptors, such as LAG-3, in addition to PD-1 are showing promising results in trials [98,99]. Future work should focus on rationally identifying optimal combinations of ICB that can promote sustained antitumour immune responses. To achieve this, fundamental insight into the molecular functions of different classes of immune checkpoint is still required to inform treatment strategies in humans.

## Future perspectives

To understand which therapeutic strategies are appropriate and for which patients, future research must answer two key questions: (i) what are the signatures of T cells in which functionality is restored during single agent/combination ICB? And (ii): what is the molecular response to ICB that underpins resistance in responding T cells? The former will help target them at the right patients, maximising clinical benefit and minimising unnecessary toxicity and the latter will inform design of rational therapy combinations.

Thorough mechanistic work in experimental models of cancer, tracking dynamic changes in T cells during ICB, will be key to answering the above questions. Translating work in mouse to human is a key challenge in the field: innovative approaches such as tumour-explant models [100] have allowed well-controlled comparisons between ICB approaches, providing valuable information regarding T-cell differentiation during therapy. This will help us understand what therapies synergise with blockade of ICs, such as PD-1, and how they might be used to augment or prolong the clinical benefit of ICB.

## Summary

- TCR signal duration and strength drive heterogeneous T-cell exhaustion.
- Stage of T-cell exhaustion dictates molecular response to ICB.
- T-cell proliferation and effector differentiation are signatures of effective ICB therapy.
- Poor pre-existing immune response and homologous terminal exhaustion can both drive primary resistance to ICB.
- ICB may drive acquired resistance via enhanced negative feedback.

## Abbreviations

APC: antigen-presenting cell
CDR3: complimentary determining region 3
DC: dendritic cell
ICB: immune checkpoint blockade
IFNγ: Interferon-gamma
LAG-3: lymphocyte-activation gene 3
LCMV: lymphocytic choriomeningitis virus
MHC: major histocompatibility complex
PD-1: programmed cell death 1
PD-L1: ligand for PD-1
pMHC: peptide-loaded MHC
TCF: T-cell factor 1
TCR: T-cell receptor
TIGIT: T-cell immunoreceptor with Ig and ITIM domains
TIL: tumour-infiltrating lymphocyte
TIM: T-cell immunoglobulin and mucin domain 3
TNF: tumour necrosis factor-alpha

## References

[1] Beal A.M., Anikeeva N., Varma R., Cameron T.O., Vasiliver-Shamis G., Norris P.J. et al. (2009) Kinetics of early T cell receptor signaling regulate the pathway of lytic granule delivery to the secretory domain. Immunity 31, 632–642 10.1016/j.immuni.2009.09.004 19833088 PMC2778196

[2] Thibodeau J., Bourgeois-Daigneault M.C. and Lapointe R. (2012) Targeting the MHC Class II antigen presentation pathway in cancer immunotherapy. Oncoimmunology 1, 908–916 10.4161/onci.21205 23162758 PMC3489746

[3] Wosen J.E., Mukhopadhyay D., MacAubas C. and Mellins E.D. (2018) Epithelial MHC class II expression and its role in antigen presentation in the gastrointestinal and respiratory tracts. Front. Immunol. 9, 1–14 10.3389/fimmu.2018.02144 30319613 PMC6167424

[4] Cachot A., Bilous M., Liu Y.C., Li X., Saillard M., Cenerenti M. et al. (2021) Tumor-specific cytolytic CD4 T cells mediate immunity against human cancer. Sci. Adv. 7, 1–19 10.1126/sciadv.abe3348 PMC7909889 33637530

[5] Moran A.E., Holzapfel K.L., Xing Y., Cunningham N.R., Maltzman J.S., Punt J. et al. (2011) T cell receptor signal strength in Treg and iNKT cell development demonstrated by a novel fluorescent reporter mouse. J. Exp. Med. 208, 1279–1289 10.1084/jem.20110308 21606508 PMC3173240

[6] Elliot T.A.E., Jennings E.K., Lecky D.A.J., Rouvray S., Mackie G.M., Scarfe L. et al. (2022) Nur77-Tempo mice reveal T cell steady state antigen recognition. Discovery Immunol. 1, kyac009 10.1093/discim/kyac009 36704407 PMC7614040

[7] Snook J.P., Kim C. and Williams M.A. (2018) TCR signal strength controls the differentiation of CD4+ effector and memory T cells. Sci. Immunol. 3, 1–13 10.1126/sciimmunol.aas9103 PMC6126666 30030369

[8] Solouki S., Huang W., Elmore J., Limper C., Huang F. and August A.T.C.R. (2020) Signal strength and antigen affinity regulate CD8 + memory T cells. J. Immunol. 205, 1217–1227 10.4049/jimmunol.1901167 32759295 PMC8104072

[9] Huang X. and Yang Y. (2006) The fate of effector CD8 T cells in vivo is controlled by the duration of antigen stimulation. Immunology 118, 361–371 10.1111/j.1365-2567.2006.02381.x 16827897 PMC1782300

[10] King C.G., Koehli S., Hausmann B., Schmaler M., Zehn D. and Palmer E. (2012) T cell affinity regulates asymmetric division, effector cell differentiation, and tissue pathology. Immunity 37, 709–720 10.1016/j.immuni.2012.06.021 23084359 PMC3622938

[11] Richard A.C., Lun A.T.L., Lau W.W.Y., Göttgens B., Marioni J.C. and Griffiths G.M. (2018) T cell cytolytic capacity is independent of initial stimulation strength. Nat. Immunol. 19, 849–858 10.1038/s41590-018-0160-9 30013148 PMC6300116

[12] Frazer G.L., Gawden-Bone C.M., Dieckmann N.M.G., Asano Y. and Griffiths G.M. (2021) Signal strength controls the rate of polarization within ctls during killing. J. Cell Biol. 220, e202104093 10.1083/jcb.202104093 34292303 PMC8302442

[13] Hogquist K.A., Baldwin T.A. and Jameson S.C. (2005) Central tolerance: learning self-control in the thymus. Nat. Rev. Immunol. 5, 772–782 10.1038/nri1707 16200080

[14] Shah S., Al-Omari A., Cook K.W., Paston S.J., Durrant L.G. and Brentville V.A. (2023) What do cancer-specific T cells ‘see’? Discovery Immunol. 2, kyac011 10.1093/discim/kyac011 PMC10917189 38567060

[15] Schumacher T.N. and Schreiber R.D. (2015) Neoantigens in cancer immunotherapy. Science 348, 69–74 10.1126/science.aaa4971 25838375

[16] Alexandrov L.B., Kim J., Haradhvala N.J., Huang M.N., Tian Ng A.W., Wu Y. et al. (2020) The repertoire of mutational signatures in human cancer. Nature 578, 94–101 10.1038/s41586-020-1943-3 32025018 PMC7054213

[17] Germano G., Lamba S., Rospo G., Barault L., Magri A., Maione F. et al. (2017) Inactivation of DNA repair triggers neoantigen generation and impairs tumour growth. Nature 552, 116–120 10.1038/nature24673 29186113

[18] Chen L. and Flies D.B. (2013) Molecular mechanisms of T cell co-stimulation and co-inhibition. Nat. Rev. Immunol. 13, 227–242 10.1038/nri3405 23470321 PMC3786574

[19] Hui E., Cheung J., Zhu J., Su X., Taylor M.J., Wallweber H.A. et al. (2017) T cell costimulatory receptor CD28 is a primary target for PD-1-mediated inhibition. Science 355, 1428–1433 10.1126/science.aaf1292 28280247 PMC6286077

[20] Kamphorst A.O., Wieland A., Nasti T., Yang S., Zhang R., Barber D.L. et al. (2017) Rescue of exhausted CD8 T cells by PD-1-targeted therapies is CD28-dependent. Science 355, 1423–1427 10.1126/science.aaf0683 28280249 PMC5595217

[21] Mizuno R., Sugiura D., Shimizu K., Maruhashi T., Watada M., Okazaki IL-Mi et al. (2019) PD-1 primarily targets TCR signal in the inhibition of functional T cell activation. Front. Immunol. 10, 1–14 10.3389/fimmu.2019.00630 31001256 PMC6455061

[22] Bevington S.L., Ng S.T.H., Britton G.J., Keane P., Wraith D.C. and Cockerill P.N. (2020) Chromatin priming renders T cell tolerance-associated genes sensitive to activation below the signaling threshold for immune response genes. Cell Rep. 31, 107748 10.1016/j.celrep.2020.107748 32521273 PMC7296351

[23] Elliot T.A.E., Jennings E.K., Lecky D.A.J., Thawait N., Flores-Langarica A., Copland A. et al. (2021) Antigen and checkpoint receptor engagement recalibrates T cell receptor signal strength. Immunity 54, 2481.e6–2496.e6 10.1016/j.immuni.2021.08.020 34534438 PMC8585507

[24] Wei S.C., Duffy C.R. and Allison J.P. (2018) Fundamental mechanisms of immune checkpoint blockade therapy. Cancer Discov. 8, 1069–1086 10.1158/2159-8290.CD-18-0367 30115704

[25] Maleki Vareki S. (2018) High and low mutational burden tumors versus immunologically hot and cold tumors and response to immune checkpoint inhibitors. J. Immunother. Cancer 6, 157 10.1186/s40425-018-0479-7 30587233 PMC6307306

[26] Schoenfeld A.J. and Hellmann M.D. (2020) Acquired resistance to immune checkpoint inhibitors. Cancer Cell. 37, 443–455 10.1016/j.ccell.2020.03.017 32289269 PMC7182070

[27] Litchfield K., Reading J.L., Puttick C., Thakkar K., Abbosh C., Bentham R. et al. (2021) Meta-analysis of tumor- and T cell-intrinsic mechanisms of sensitization to checkpoint inhibition. Cell 184, 596.e14–614.e14 10.1016/j.cell.2021.01.002 33508232 PMC7933824

[28] de Vries N.L., van de Haar J., Veninga V., Chalabi M., Ijsselsteijn M.E., van der Ploeg M. et al. (2023) γδ T cells are effectors of immunotherapy in cancers with HLA class I defects. Nature 613, 743–750 10.1038/s41586-022-05593-1 36631610 PMC9876799

[29] Zaretsky J.M., Garcia-Diaz A., Shin D.S., Escuin-Ordinas H., Hugo W., Hu-Lieskovan S. et al. (2016) Mutations associated with acquired resistance to PD-1 blockade in melanoma. N. Engl. J. Med. 375, 819–829 10.1056/NEJMoa1604958 27433843 PMC5007206

[30] Riaz N., Havel J.J., Makarov V., Desrichard A., Sharfman W.H., Bhatia S. et al. (2017) Tumor and microenvironment evolution during immunotherapy with nivolumab. Cell 171, 934–949 10.1016/j.cell.2017.09.028 29033130 PMC5685550

[31] Pietrobon V. and Marincola F.M. (2021) Hypoxia and the phenomenon of immune exclusion. J. Transl. Med. 19, 9 10.1186/s12967-020-02667-4 33407613 PMC7788724

[32] Hegde P.S. and Chen D.S. (2020) Top 10 challenges in cancer immunotherapy. Immunity 52, 17–35 10.1016/j.immuni.2019.12.011 31940268

[33] Gabrilovich D.I. (2017) Myeloid-derived suppressor cells. Cancer Immunol. Res. 5, 3–8 10.1158/2326-6066.CIR-16-0297 28052991 PMC5426480

[34] Scott A.C., Dündar F., Zumbo P., Chandran S.S., Klebanoff C.A., Shakiba M. et al. (2019) TOX is a critical regulator of tumour-specific T cell differentiation. Nature 571, 270–274 10.1038/s41586-019-1324-y 31207604 PMC7698992

[35] Barber D.L., Wherry E.J., Masopust D., Zhu B., Allison J.P., Sharpe A.H. et al. (2006) Restoring function in exhausted CD8 T cells during chronic viral infection. Nature 439, 682–687 10.1038/nature04444 16382236

[36] Wherry E.J. (2011) T cell exhaustion. Nat. Immunol. 12, 492–499 10.1038/ni.2035 21739672

[37] Schietinger A., Philip M., Krisnawan V.E., Chiu E.Y., Delrow J.J., Basom R.S. et al. (2016) Tumor-specific T cell dysfunction is a dynamic antigen-driven differentiation program initiated early during tumorigenesis. Immunity 45, 389–401 10.1016/j.immuni.2016.07.011 27521269 PMC5119632

[38] Singer M., Wang C., Cong L., Marjanovic N.D., Kowalczyk M.S., Zhang H. et al. (2016) A distinct gene module for dysfunction uncoupled from activation in tumor-infiltrating T cells. Cell 166, 1500.e9–1511.e9 10.1016/j.cell.2016.08.052 27610572 PMC5019125

[39] Utzschneider D.T., Charmoy M., Chennupati V., Pousse L., Ferreira D.P., Calderon-Copete S. et al. (2016) T cell factor 1-expressing memory-like CD8+ T cells sustain the immune response to chronic viral infections. Immunity 45, 415–427 10.1016/j.immuni.2016.07.021 27533016

[40] Im S.J., Hashimoto M., Gerner M.Y., Lee J., Kissick H.T., Burger M.C. et al. (2016) Defining CD8+ T cells that provide the proliferative burst after PD-1 therapy. Nature 537, 417–421 10.1038/nature19330 27501248 PMC5297183

[41] Brummelman J., Mazza E.M.C., Alvisi G., Colombo F.S., Grilli A., Mikulak J. et al. (2018) High-dimensional single cell analysis identifies stemlike cytotoxic CD8+T cells infiltrating human tumors. J. Exp. Med. 215, 2520–2535 10.1084/jem.20180684 30154266 PMC6170179

[42] Miller B.C., Sen D.R., Al Abosy R., Bi K., Virkud Y.V., LaFleur M.W. et al. (2019) Subsets of exhausted CD8+ T cells differentially mediate tumor control and respond to checkpoint blockade. Nat. Immunol. 20, 326–336 10.1038/s41590-019-0312-6 30778252 PMC6673650

[43] Mazet J.M., Mahale J.N., Tong O., Watson R.A., Lechuga-Vieco A.V., Pirgova G. et al. (2023) IFNγ signaling in cytotoxic T cells restricts anti-tumor responses by inhibiting the maintenance and diversity of intra-tumoral stem-like T cells. Nat. Commun. 14, 321 10.1038/s41467-023-35948-9 36658158 PMC9852295

[44] Utzschneider D.T., Legat A., Fuertes Marraco S.A., Carrié L., Luescher I., Speiser D.E. et al. (2013) T cells maintain an exhausted phenotype after antigen withdrawal and population reexpansion. Nat. Immunol. 14, 603–610 10.1038/ni.2606 23644506

[45] Sekine T., Perez-Potti A., Nguyen S., Gorin J.B., Wu V.H., Gostick E. et al. (2020) TOX is expressed by exhausted and polyfunctional human effector memory CD8+ T cells. Sci. Immunol. 5, 1–15 10.1126/sciimmunol.aba7918 32620560

[46] Khan O., Giles J.R., McDonald S., Manne S., Ngiow S.F., Patel K.P. et al. (2019) TOX transcriptionally and epigenetically programs CD8+ T cell exhaustion. Nature 571, 211–218 10.1038/s41586-019-1325-x 31207603 PMC6713202

[47] Chihara N., Madi A., Kondo T., Zhang H., Acharya N., Singer M. et al. (2018) Induction and transcriptional regulation of the co-inhibitory gene module in T cells. Nature 558, 454–459 10.1038/s41586-018-0206-z 29899446 PMC6130914

[48] Tsui C., Kretschmer L., Rapelius S., Gabriel S.S., Chisanga D., Knöpper K. et al. (2022) MYB orchestrates T cell exhaustion and response to checkpoint inhibition. Nature 609, 354–360 10.1038/s41586-022-05105-1 35978192 PMC9452299

[49] Liu X., Wang Y., Lu H., Li J., Yan X., Xiao M. et al. (2019) Genome-wide analysis identifies NR4A1 as a key mediator of T cell dysfunction. Nature 567, 525–529 10.1038/s41586-019-0979-8 30814730 PMC6507425

[50] Chen J., López-Moyado I.F., Seo H., Lio C.W.J., Hempleman L.J., Sekiya T. et al. (2019) NR4A transcription factors limit CAR T cell function in solid tumours. Nature 567, 530–534 10.1038/s41586-019-0985-x 30814732 PMC6546093

[51] Martinez G.J., Pereira R.M., Äijö T., Kim E.Y., Marangoni F., Pipkin M.E. et al. (2015) The transcription factor NFAT promotes exhaustion of activated CD8+ T cells. Immunity 42, 265–278 10.1016/j.immuni.2015.01.006 25680272 PMC4346317

[52] Utzschneider D.T., Gabriel S.S., Chisanga D., Gloury R., Gubser P.M., Vasanthakumar A. et al. (2020) Early precursor T cells establish and propagate T cell exhaustion in chronic infection. Nat. Immunol. 21, 1256–1266 10.1038/s41590-020-0760-z 32839610

[53] Philip M., Fairchild L., Sun L., Horste E.L., Camara S., Shakiba M. et al. (2017) Chromatin states define tumour-specific T cell dysfunction and reprogramming. Nature 545, 452–456 10.1038/nature22367 28514453 PMC5693219

[54] Khan O., Giles J.R., McDonald S., Manne S., Ngiow S.F., Patel K.P. et al. (2019) TOX transcriptionally and epigenetically programs CD8+ T cell exhaustion. Nature 571, 211–218 10.1038/s41586-019-1325-x 31207603 PMC6713202

[55] Zhao M., Kiernan C.H., Stairiker C.J., Hope J.L., Leon L.G., van Meurs M. et al. (2020) Rapid in vitro generation of bona fide exhausted CD8+ T cells is accompanied by Tcf7 promotor methylation. PLoS Pathog. 16, 1–27 10.1371/journal.ppat.1008555 PMC7340326 32579593

[56] Blackburn S.D., Shin H., Haining W.N., Zou T., Workman C.J., Polley A. et al. (2009) Coregulation of CD8+ T cell exhaustion by multiple inhibitory receptors during chronic viral infection. Nat. Immunol. 10, 29–37 10.1038/ni.1679 19043418 PMC2605166

[57] Han H.S., Jeong S., Kim H., Kim H.D., Kim A.R., Kwon M. et al. (2021) TOX-expressing terminally exhausted tumor-infiltrating CD8+ T cells are reinvigorated by co-blockade of PD-1 and TIGIT in bladder cancer. Cancer Lett. 499, 137–147 10.1016/j.canlet.2020.11.035 33249194

[58] Knuschke T., Kollenda S., Wenzek C., Zelinskyy G., Steinbach P., Dittmer U. et al. (2021) A combination of anti-pd-l1 treatment and therapeutic vaccination facilitates improved retroviral clearance via reactivation of highly exhausted t cells. mBio 12, 1–16 10.1128/mBio.02121-20 PMC7858051 33531395

[59] Guo Y., Xie Y.Q., Gao M., Zhao Y., Franco F., Wenes M. et al. (2021) Metabolic reprogramming of terminally exhausted CD8+ T cells by IL-10 enhances anti-tumor immunity. Nat. Immunol. 22, 746–756 10.1038/s41590-021-00940-2 34031618 PMC7610876

[60] Daniel B., Yost K.E., Hsiung S., Sandor K., Xia Y., Qi Y. et al. (2022) Divergent clonal differentiation trajectories of T cell exhaustion. Nat. Immunol. 23, 1614–1627 10.1038/s41590-022-01337-5 36289450 PMC11225711

[61] Shakiba M., Zumbo P., Espinosa-Carrasco G., Menocal L., Dündar F., Carson S.E. et al. (2021) TCR signal strength defines distinct mechanisms of T cell dysfunction and cancer evasion. J. Exp. Med. 219, e20201966 10.1084/jem.20201966 34935874 PMC8704919

[62] Shimizu K., Sugiura D., Okazaki IL-Mi, Maruhashi T., Takegami Y., Cheng C. et al. (2020) PD-1 imposes qualitative control of cellular transcriptomes in response to T cell activation. Mol. Cell. 77, 937.e6–950.e6 10.1016/j.molcel.2019.12.012 31926851

[63] Spitzer M.H., Carmi Y., Reticker-Flynn N.E., Kwek S.S., Madhireddy D., Martins M.M. et al. (2017) Systemic immunity is required for effective cancer immunotherapy. Cell 168, 487.e15–502.e15 10.1016/j.cell.2016.12.022 28111070 PMC5312823

[64] Weber E.W., Parker K.R., Sotillo E., Lynn R.C., Anbunathan H., Lattin J. et al. (2021) Transient rest restores functionality in exhausted CAR-T cells through epigenetic remodeling. Science 372, eaba1786 10.1126/science.aba1786 33795428 PMC8049103

[65] Li Z., Tuong Z.K., Dean I., Willis C., Gaspal F., Fiancette R. et al. (2022) *In vivo* labeling reveals continuous trafficking of TCF-1+ T cells between tumor and lymphoid tissue. J. Exp. Med. 219, e20210749 10.1084/jem.20210749 35472220 PMC9048291

[66] Kennedy B.C., Dean I. and Withers D.R. (2023) Migration of stem-like CD8 T cells between tissue microenvironments underpins successful anti- tumour immune responses. Discovery Immunol. 2, kyad004 10.1093/discim/kyad004 37008996 PMC10052398

[67] Connolly K.A., Kuchroo M., Venkat A., Khatun A., Wang J., William I. et al. (2021) A reservoir of stem-like CD8 + T cells in the tumor-draining lymph node preserves the ongoing antitumor immune response. Sci. Immunol. 6, 7836 10.1126/sciimmunol.abg7836 PMC8593910 34597124

[68] Prokhnevska N., Cardenas M.A., Valanparambil R.M., Sobierajska E., Barwick B.G., Jansen C. et al. (2023) CD8+ T cell activation in cancer comprises an initial activation phase in lymph nodes followed by effector differentiation within the tumor. Immunity 56, 107.e5–124.e5 10.1016/j.immuni.2022.12.002 36580918 PMC10266440

[69] Siddiqui I., Schaeuble K., Chennupati V., Fuertes Marraco S.A., Calderon-Copete S., Pais Ferreira D. et al. (2019) Intratumoral Tcf1 + PD-1 + CD8 + T cells with stem-like properties promote tumor control in response to vaccination and checkpoint blockade immunotherapy. Immunity 50, 195.e10–211.e10 10.1016/j.immuni.2018.12.021 30635237

[70] Kreiter S., Vormehr M., Van De Roemer N., Diken M., Löwer M., Diekmann J. et al. (2015) Mutant MHC class II epitopes drive therapeutic immune responses to cancer. Nature 520, 692–696 10.1038/nature14426 25901682 PMC4838069

[71] Linnemann C., Van Buuren M.M., Bies L., Verdegaal E.M.E., Schotte R., Calis J.J.A. et al. (2015) High-throughput epitope discovery reveals frequent recognition of neo-antigens by CD4+ T cells in human melanoma. Nat. Med. 21, 81–85 10.1038/nm.3773 25531942

[72] Alspach E., Lussier D.M., Miceli A.P., Kizhvatov I., DuPage M., Luoma A.M. et al. (2019) MHC-II neoantigens shape tumour immunity and response to immunotherapy. Nature 574, 696–701 10.1038/s41586-019-1671-8 31645760 PMC6858572

[73] Laidlaw B.J., Craft J.E. and Kaech S.M. (2016) The multifaceted role of CD4+ T cells in CD8+ T cell memory. Nat. Rev. Immunol. 16, 102–111 10.1038/nri.2015.10 26781939 PMC4860014

[74] Mortara L., Castellani P., Meazza R., Tosi G., De Lerma Barbaro A., Procopio F.A. et al. (2006) CIITA-induced MHC class II expression in mammary adenocarcinoma leads to a Th1 polarization of the tumor microenvironment, tumor rejection, and specific antitumor memory. Clin. Cancer Res. 12, 3435–3443 10.1158/1078-0432.CCR-06-0165 16740768

[75] Burger M.L., Cruz A.M., Crossland G.E., Gaglia G., Ritch C.C., Blatt S.E. et al. (2021) Antigen dominance hierarchies shape TCF1+ progenitor CD8 T cell phenotypes in tumors. Cell 184, 4996.e26–5014.e26 10.1016/j.cell.2021.08.020 34534464 PMC8522630

[76] Koyama S., Akbay E.A., Li Y.Y., Herter-sprie G.S., Buczkowski K.A., Richards W.G. et al. (2016) Adaptive resistance to therapeutic PD-1 blockade is associated with upregulation of alternative immune checkpoints. Nat. Commun. 7, 1–9 10.1038/ncomms10501 PMC4757784 26883990

[77] Sakuishi K., Apetoh L., Sullivan J.M., Blazar B.R., Kuchroo V.K. and Anderson A.C. (2010) Targeting Tim-3 and PD-1 pathways to reverse T cell exhaustion and restore anti-tumor immunity. J. Exp. Med. 207, 2187–2194 10.1084/jem.20100643 20819927 PMC2947065

[78] Woo S.R., Turnis M.E., Goldberg M.V., Bankoti J., Selby M., Nirschl C.J. et al. (2012) Immune inhibitory molecules LAG-3 and PD-1 synergistically regulate T-cell function to promote tumoral immune escape. Cancer Res. 72, 917–927 10.1158/0008-5472.CAN-11-1620 22186141 PMC3288154

[79] Vafaei S., Zekiy A.O., Khanamir R.A., Zaman B.A., Ghayourvahdat A., Azimizonuzi H. et al. (2022) Combination therapy with immune checkpoint inhibitors (ICIs); a new frontier. Cancer Cell Int. 22, 1–27 10.1186/s12935-021-02407-8 34980128 PMC8725311

[80] Dammeijer F., Van Gulijk M., Mulder E.E., Verhoef C., Van Hall T. and Aerts J.G. (2020) The PD-1/PD-L1-checkpoint restrains T cell immunity in tumor-draining lymph nodes article. Cancer Cell. 38, 1–16 10.1016/j.ccell.2020.09.001 33007259

[81] Yost K.E., Satpathy A.T., Wells D.K., Qi Y., Wang C., Kageyama R. et al. (2019) Clonal replacement of tumor-specific T cells following PD-1 blockade. Nat. Med. 25, 1251–1259 10.1038/s41591-019-0522-3 31359002 PMC6689255

[82] Watson R.A., Tong O., Cooper R., Taylor C.A., Sharma P.K., Verge De Los Aires A. et al. (2021) Immune checkpoint blockade sensitivity and progression-free survival associates with baseline CD8 + T cell clone size and cytotoxicity. Sci. Immunol. 6, 8825 10.1126/sciimmunol.abj8825 PMC7612602 34597125

[83] Fairfax B.P., Taylor C.A., Watson R.A., Nassiri I., Danielli S., Fang H. et al. (2020) Peripheral CD8+ T cell characteristics associated with durable responses to immune checkpoint blockade in patients with metastatic melanoma. Nat. Med. 26, 193–199 10.1038/s41591-019-0734-6 32042196 PMC7611047

[84] Caushi J.X., Zhang J., Ji Z., Vaghasia A., Zhang B., Hsiue E.H.C. et al. (2021) Transcriptional programs of neoantigen-specific TIL in anti-PD-1-treated lung cancers. Nature 596, 126–132 10.1038/s41586-021-03752-4 34290408 PMC8338555

[85] Fehlings M., Kim L., Guan X., Yuen K., Tafazzol A., Sanjabi S. et al. (2022) Single-cell analysis reveals clonally expanded tumor-associated CD57^+^ CD8 T cells are enriched in the periphery of patients with metastatic urothelial cancer responding to PD-L1 blockade. J. Immunother. Cancer 10, e004759 10.1136/jitc-2022-004759 35981786 PMC9394212

[86] Tumeh P.C., Harview C.L., Yearley J.H., Shintaku I.P., Taylor E.J.M., Robert L. et al. (2014) PD-1 blockade induces responses by inhibiting adaptive immune resistance. Nature 515, 568–571 10.1038/nature13954 25428505 PMC4246418

[87] Ayyoub M., Pignon P., Classe J.M., Odunsi K. and Valmori D. (2013) CD4+ T effectors specific for the tumor antigen NY-ESO-1 are highly enriched at ovarian cancer sites and coexist with, but are distinct from, tumor-associated Treg. Cancer Immunol. Res. 1, 303–308 10.1158/2326-6066.CIR-13-0062-T 24777968

[88] Balança C.C., Salvioni A., Scarlata C.M., Michelas M., Martinez-Gomez C., Gomez-Roca C. et al. (2021) PD-1 blockade restores helper activity of tumor-infiltrating, exhausted PD-1hiCD39+ CD4 T cells. JCI Insight 6, e142513 10.1172/jci.insight.142513 33332284 PMC7934837

[89] Simoni Y., Becht E., Fehlings M., Loh C.Y., Koo S.L., Teng K.W.W. et al. (2018) Bystander CD8+ T cells are abundant and phenotypically distinct in human tumour infiltrates. Nature 557, 575–579, 10.1038/s41586-018-0130-2 29769722

[90] Oliveira G., Stromhaug K., Klaeger S., Kula T., Frederick D.T., Le P.M. et al. (2021) Phenotype, specificity and avidity of antitumour CD8+ T cells in melanoma. Nature 596, 119–125 10.1038/s41586-021-03704-y 34290406 PMC9187974

[91] Jansen C.S., Prokhnevska N., Master V.A., Sanda M.G., Carlisle J.W., Bilen M.A. et al. (2019) An intra-tumoral niche maintains and differentiates stem-like CD8 T cells. Nature 576, 465–470 10.1038/s41586-019-1836-5 31827286 PMC7108171

[92] Sade-Feldman M., Yizhak K., Bjorgaard S.L., Ray J.P., de Boer C.G., Jenkins R.W. et al. (2018) Defining T cell states associated with response to checkpoint immunotherapy in melanoma. Cell 175, 998.e20–1013.e20 10.1016/j.cell.2018.10.038 30388456 PMC6641984

[93] Blank C.U., Rozeman E.A., Fanchi L.F., Sikorska K., van de Wiel B., Kvistborg P. et al. (2018) Neoadjuvant versus adjuvant ipilimumab plus nivolumab in macroscopic stage III melanoma. Nat. Med. 24, 1655–1661 10.1038/s41591-018-0198-0 30297911

[94] Chalabi M., Fanchi L.F., Dijkstra K.K., Van den Berg J.G., Aalbers A.G., Sikorska K. et al. (2020) Neoadjuvant immunotherapy leads to pathological responses in MMR-proficient and MMR-deficient early-stage colon cancers. Nat. Med. 26, 566–576 10.1038/s41591-020-0805-8 32251400

[95] Huang A.C., Postow M.A., Orlowski R.J., Mick R., Bengsch B., Manne S. et al. (2017) T-cell invigoration to tumour burden ratio associated with anti-PD-1 response. Nature 545, 60–65 10.1038/nature22079 28397821 PMC5554367

[96] Gettinger S., Choi J., Hastings K., Truini A., Datar I., Sowell R. et al. (2017) Impaired HLA class I antigen processing and presentation as a mechanism of acquired resistance to immune checkpoint inhibitors in lung cancer. Cancer Discov. 7, 1420–1435 10.1158/2159-8290.CD-17-0593 29025772 PMC5718941

[97] Kakavand H., Jackett L.A., Menzies A.M., Gide T.N., Carlino M.S., Saw R.P.M. et al. (2017) Negative immune checkpoint regulation by VISTA: a mechanism of acquired resistance to anti-PD-1 therapy in metastatic melanoma patients. Mod. Pathol. 30, 1666–1676 10.1038/modpathol.2017.89 28776578

[98] Tawbi H.A., Schadendorf D., Lipson E.J., Ascierto P.A., Matamala L., Castillo Gutiérrez E. et al. (2022) Relatlimab and nivolumab versus nivolumab in untreated advanced melanoma. N. Engl. J. Med. 386, 24–34 10.1056/NEJMoa2109970 34986285 PMC9844513

[99] Huuhtanen J., Kasanen H.H., Peltola K., Lönnberg T., Glumoff V., Brück O. et al. (2023) Single-cell characterization of anti-LAG-3 and anti-PD-1 treatment in melanoma patients. J. Clin. Invest. 113, e164809 10.1172/JCI164809 36719749 PMC10014104

[100] Voabil P., de Bruijn M., Roelofsen L.M., Hendriks S.H., Brokamp S., van den Braber M. et al. (2021) An ex vivo tumor fragment platform to dissect response to PD-1 blockade in cancer. Nat. Med. 27, 1250–1261 10.1038/s41591-021-01398-3 34239134

